# Response to letter to the editor: On methodological approaches for non‐invasive characterization of muscle oxygen uptake recovery kinetics

**DOI:** 10.1113/EP094538

**Published:** 2026-11-09

**Authors:** Edward Z. Pelka, B. Ryan Davis, John McDaniel

**Affiliations:** ^1^ Exercise Science and Exercise Physiology Program Kent State University Kent Ohio USA; ^2^ Department of Kinesiology University of North Georgia Dahlonega Georgia USA; ^3^ Louis Stokes Cleveland Veterans Affairs Medical Center Cleveland Ohio USA

1

To the Editor,

We thank Marciano and Rossiter (2026) for their interest in our recent investigation and welcome discussion regarding methodological approaches for near‐infrared spectroscopy (NIRS)‐derived assessment of skeletal muscle oxidative capacity. We wish to clarify the use of conventional time‐based protocols and the ∼15s interval between exercise cessation and initiation of intermittent arterial occlusions in our investigation (Pelka et al., 2026).

Initially, Marciano and Rossiter suggest that not assessing muscle oxygen consumption ($m{\dot{V}}_{O_{2}}$) immediately after cessation of exercise misses the most dynamic portion of recovery. In addition, they contend that the measurements obtained later in recovery contain limited kinetic information, a limitation that might negatively affect the validity of the recovery rate constant (*k*). We agree that earlier measurements contain greater absolute changes in $m{\dot{V}}_{O_{2}}$ and might provide additional information for estimation of the rate constant. However, when $m{\dot{V}}_{O_{2}}$ recovery is adequately described by a monoexponential function, a single *k* characterizes the rate of recovery. Thus, omission of the initial portion of the curve might reduce the amount of information available to estimate *k* but does not inherently change the estimated rate constant.

With conventional time‐based occlusion protocols, a 15s interval corresponds roughly to the first data point rather than exclusion of a substantial portion of the early dynamic recovery response. This interval used in our investigation was intentional and was incorporated to allow postexercise perfusion and avoid low initial $m{\dot{V}}_{O_{2}}$ values, as observed in previous reports, including but not limited to Beever et al. (2020) and Pilotto et al. (2022). Beever et al. (2020) reported that the slope from the first arterial occlusion frequently required removal because it was equal to or lower than the second slope. They proposed that this response could reflect O_2_ limitation or a contribution of glycolysis to phosphocreatine recovery during the first seconds following exercise (Beever et al., 2020). Accordingly, in their analysis initial aberrant values were removed before curve fitting; this exclusion is evident in their representative data (Beever et al., 2020: fig. 1A,B). Likewise, Pilotto et al. (2022) reported removal of low initial tissue saturation index (TSI) values before curve fitting during saturation‐controlled protocols. These low initial slopes are apparent in their representative data (Pilotto et al., 2022: fig. 2C). If inclusion of the earliest recovery measurements were necessary for a valid estimate of *k*, exclusion of those data points during curve fitting would raise a similar concern, especially if removal of data points is not consistent across subjects or treatments.

Our approach of waiting for 15s before the first occlusion is not unique to our investigation. Tripp et al. (2024) used NIRS to assess oxidative capacity of the vastus lateralis and deliberately allowed 15 s after 40% maximal voluntary contractions and 30 s after maximal voluntary contractions before initiating repeated arterial occlusions. The 15 s interval was intended to allow for the immediate transition from exercise to recovery during the period in which low initial $m{\dot{V}}_{O_{2}}$ measurements have previously been observed, rather than excluding a substantial portion of the recovery data.

We also caution against interpreting the absolute *k* of ∼1.0 min^−1^ reported in our investigation as evidence that the 15 s interval lowered *k*. Although values near 2.0 min^−1^ have been reported in healthy individuals, the lower *k* cannot be attributed to the 15 s interval alone. Notably, Tripp et al. (2024) used the same 15 s interval following contractions of the vastus lateralis and reported a mean *k* of 2.68 ± 0.75 min^−1^ (Tripp et al., 2024). Therefore, the absolute *k* observed in our investigation cannot, in isolation, be used as evidence that the 15 s interval systematically underestimates *k*.

We acknowledge the potential advantages of saturation‐controlled approaches for increasing early sampling frequency and controlling tissue oxygenation. However, whether this represents an optimal approach remains to be established. Indeed, methodological concerns regarding this approach have been raised (Manferdelli et al., 2022). Manferdelli et al. (2022), commenting specifically on the saturation‐controlled approach described by Pilotto et al. (2022), noted that varying the number, timing and duration of intermittent arterial occlusions to maintain a predetermined TSI range might introduce concerns regarding standardization and potentially influence calculation of *k* (Manferdelli et al., 2022). Although saturation‐controlled protocols provide greater control over O_2_ availability, manipulating tissue oxygenation during recovery does not reproduce the same physiological conditions of postexercise recovery. Thus, saturation‐controlled and standardized time‐based protocols involve different methodological considerations, and further work is needed to compare the validity and reliability of these approaches. We believe that greater clarity and transparency in reporting methodological and analytical procedures are needed to facilitate comparisons across studies and continued refinement of NIRS‐derived assessments of skeletal muscle oxidative capacity.

## AUTHOR CONTRIBUTIONS

All authors contributed to the preparation and revision of the manuscript, approved the final version, and agree to be accountable for all aspects of the work.

## CONFLICT OF INTEREST

None declared.

## FUNDING INFORMATION

None.

## GENERATIVE AI STATEMENT

No generative AI tools were used in the preparation of this manuscript.
